# Enhanced Tissue Ablation Efficiency with a Mid-Infrared Nonlinear Frequency Conversion Laser System and Tissue Interaction Monitoring Using Optical Coherence Tomography

**DOI:** 10.3390/s16050598

**Published:** 2016-04-26

**Authors:** Bongkyun Kim, Dae Yu Kim

**Affiliations:** 1 Beckman Laser Institute Korea, Dankook University, 119, Dandae-ro, Dongnam-gu, Cheonan-si, Chungnam 31116, Korea; photonicrystalfiber@gmail.com; 2 Biomedical Engineering, College of Medicine, Dankook University, 119, Dandae-ro, Dongnam-gu, Cheonan-si, Chungnam 31116, Korea

**Keywords:** near-infrared laser, mid-infrared laser system, optical coherence tomography, optical parametric oscillator, tissue ablation efficiency

## Abstract

We report development of optical parametric oscillator (OPO)-based mid-infrared laser system that utilizes a periodically poled nonlinear crystal pumped by a near-infrared (NIR) laser. We obtained a mid-infrared average output of 8 W at an injection current of 20 A from a quasi-phase-matched OPO using an external cavity configuration. Laser tissue ablation efficiency is substantially affected by several parameters, including an optical fluence rate, wavelength of the laser source, and the optical properties of target tissue. Dimensions of wavelength and radiant exposure dependent tissue ablation are quantified using Fourier domain optical coherence tomography and the ablation efficiency was compared to a non-converted NIR laser system.

## 1. Introduction

Many researchers have focused on the development of mid-infrared laser systems with stable average power over the last decade because of potential applications in spectroscopy, remote sensing, and medicine [1,2]. Since various molecules exhibit strong characteristic absorption bands at the infrared spectrum as well as the absence of mutagenic response in biological tissue, mid-infrared lasers has been widely studied for laser tissue interaction and medical applications. The purpose of minimally invasive laser tissue ablation is to remove target tissues in an efficient way while minimizing thermally induced collateral damage [3]. In efforts to exploit optimized laser parameters and wavelengths, these studies include several types of mid-infrared light sources such as free electron lasers (FELs), Carbon monoxide (CO) lasers, quantum cascade lasers (QCLs), Raman-shifted lasers, and nonlinear optical techniques [4,5,6,7,8,9,10,11,12,13,14].

In particular, 2 µm laser sources are promising candidates for laser tissue interaction because they provide superior optical absorption properties in tissue. Water, which is the largest constituent of the human body and biological tissue, is highly absorptive of 2 µm light. The favorable absorption in water makes such lasers useful for medical applications including treatments of cartilage and urologic diseases as well as laser tissue ablation. Successful laser tissue interaction studies were demonstrated in the 2 µm spectral region using thulium or holmium-based laser systems [15,16,17]. An optical parametric oscillator (OPO) is a coherent light source similar to a laser, but it is mainly based on the nonlinear frequency conversion rather than the stimulated emission in a gain medium. The OPO is an attractive way of generating the 2 µm light where typical laser systems do not exist. Moreover, the OPO can provide wideband tuning in various spectral regions for specific target chromophores.

In this study, we report development of optical parametric oscillator (OPO)-based 2 µm laser system using a periodically poled nonlinear crystal pumped by a Q-switched diode-pumped Nd:YAG laser with high average power operating with a 10 kHz repetition rate. We obtained 8 W of 2 µm average output at the injection current of 20 A from a quasi-phase-matched OPO using an external cavity configuration. In an attempt to investigate wavelength-dependent tissue ablation with an OPO-based nonlinear-frequency conversion process, our study experimentally identified an adipose tissue response to OPO-based two mid-infrared wavelengths, signal (1980 nm) and idler (2300 nm) which coincide largest water and fat absorption in near 2 µm wavelength [18]. The laser tissue ablation efficiency was also investigated. Ablation is substantially affected by several parameters including an optical fluence rate, wavelength of the laser source, and the optical properties of target tissues. Wavelength and radiant exposure dependent tissue ablation volumes were quantified and dimension of laser tissue ablation was investigated using Fourier Domain Optical Coherence Tomography (FD-OCT). We first demonstrated tissue ablation efficiency using conventional 1064 nm, 1980 nm, and newly developed 2300 nm lasers by measuring geometric dimensions in depths and diameters of laser ablation crater as well as by calculating the reduced mass after laser irradiation.

## 2. Materials and Methods

### 2.1. Mid-Infrared Generation Using Quasi-Phase Matched Nonlinear Frequency Conversion

**Figure 1:**
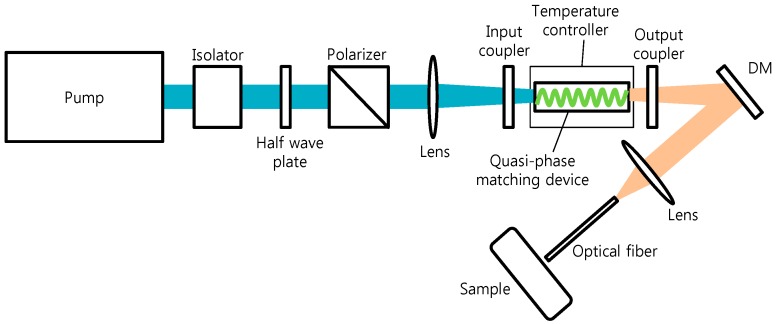
Experimental setup of OPO-based 2 micron generation for tissue ablation. DM: dichroic mirror, Pump: DPSS Nd:YAG laser (1064 nm).

For 2 µm generation, an acousto-optically Q-switched Nd:YAG laser was used for the pump for OPO cavity. The laser operated at a wavelength of 1064 nm and was optically pumped by a CW diode laser. The 1064 nm laser produced unpolarized light pulses with 40 W average power, 85 ns pulse duration, and a repetition rate of 10 kHz. The schematic of laser configuration was shown in Figure 1. A Faraday isolator was inserted between the 1064 nm pump and OPO cavity to prevent back reflection of the injected pump from the double pass OPO. The 1064 nm pump beam was apertured with a pinhole of 3 mm diameter and propagated over 1 m in order to obtain a spatially uniform beam profile. The spatial profile of the 1064 nm pump beam was approximately Gaussian with a M^2^ parameter of 2.5 and the beam diameter was measured to be approximately 3 mm. The linearly polarized output of the 1064 nm pump with 20.7 W average powers was achieved by incorporating a thin film polarizer prior to the OPO cavity. The polarized 1064 nm pump beam was focused into the PPMgSLT crystal (Oxide, Hokuto, Japan) to generate a spot size of 300 µm at the center of the nonlinear crystal using a plano-convex lens with a 15 cm focal length.

**Figure 2:**
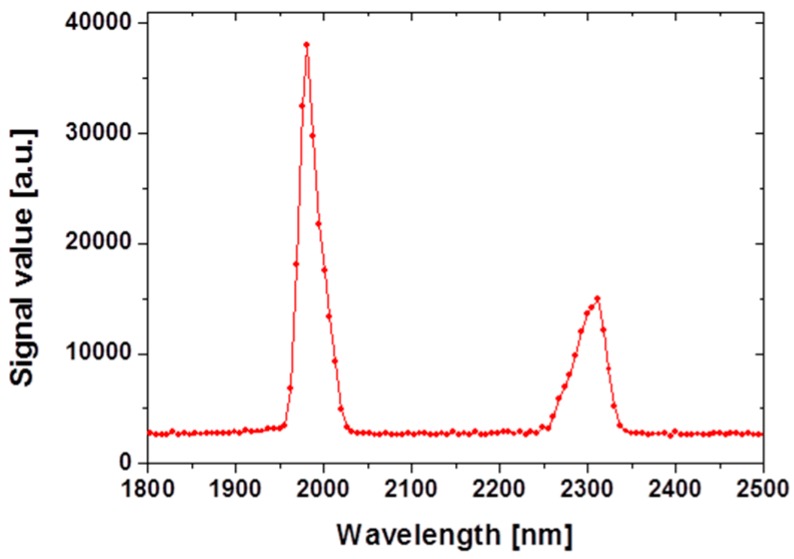
Optical spectra of the signal (1980 nm) and idler (2300 nm) waves from the OPO output.

The average power of 1064 nm pump can be adjusted by rotating the half-wave plate without changing spatial and temporal profile of the pump light. To convert the 1064 nm pump into 2 µm radiation, a 3 mm × 3 mm × 25 mm nonlinear crystal was introduced. The crystal has a grating period of 32.7 µm in the PPMgSLT substrate. A first-order quasi-phase matching process enables an effective frequency conversion from the 1064 nm pump into a 2 µm MID-IR wavelength region, resulting in the generation of signal and idler with a center wavelength of 1980 and 2300 nm, respectively, shown in Figure 2. The crystal temperature was controlled at 72 °C to satisfy a highly temperature dependent phase matching condition. The OPO cavity consisted of two plano-mirrors, input coupler, and output coupler with 45 mm separation. The nonlinear crystal was mounted at the center of the cavity. The input coupling mirror allows over 98% transmission for the 1064 nm pump and high reflectance (99%) for 2300 nm and 1980 nm. Figure 2 depicts the optical spectra of the signal and idler waves from the OPO output measured by an InGaAs spectrometer (NIRQuest; Ocean Optics, Dunedin, FL, USA). Since the output wavelength is dependent on the nonlinear crystal temperature, we controlled the crystal temperature at 72 °C. The total average output was 8 W at an injection current of 20 A from a quasi-phase-matched OPO using external cavity configuration. The stability of signal and idler output power was also measured for 60 minutes at an internal of 60 s. The root-mean-square fluctuation in the average power was 0.7% and 1.3% for signal (1980 nm) and idler (2300 nm), respectively.

### 2.2. Tissue Sample

Porcine abdominal tissues were obtained from a local abattoir and utilized for tissue ablation experiments. Samples used for ablation dimension measurement with OCT were flash frozen and then fat tissue was dissected as a flat surface (5 cm × 2 cm × 1 cm). Samples were stored in a physiologic saline solution prior to the experiment. Samples used for mass loss measurement were kept on ice without freezing and then cut into squares with initial weight of approximately 2.5 g.

### 2.3. Tissue Ablation Measurements with OCT

**Figure 3:**
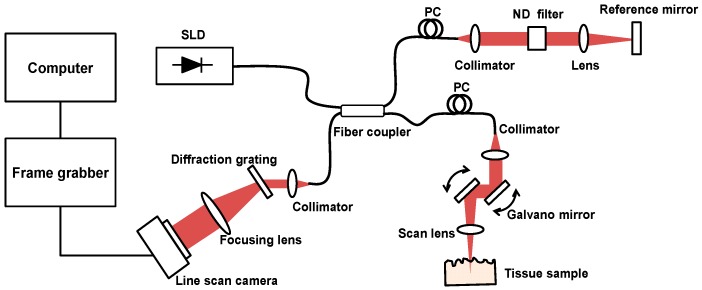
A schematic of OCT system. SLD: superluminescent diode, PC: polarization controller, ND: neutral density filter.

Signal and idler with emission wavelength of 1980 and 2300 nm, respectively, from the doubly resonant OPO system were used in the experiment. Laser irradiation of tissue was delivered by a 400 µm silica multimode fiber with predefined optical average power. Optical power at the end of the cleaved fiber was measured by a model PM10 optical power meter (Thorlabs, Inc., Newton, NJ, USA). Tissue samples (n > 10) were irradiated for measurements at each wavelength per parameter set. To investigate morphological changes and quantitative dimensions in ablation crater, spectral domain optical coherence tomography (SD-OCT) was used. OCT is a noninvasive optical imaging method applied to a wide range of medical fields including ophthalmology, dermatology, cardiology, and gastroenterology [19,20,21,22]. By integrating low-coherence interferometry and confocal laser scanning technique, it can perform cross-sectional visualization of the internal structure of tissue with a penetration depth of few mm and an axial resolution of ~15 μm [23,24,25]. OCT provides high resolution images that correlate well with histology to identify morphologic changes within the tissue. To investigate wavelength dependent ablation profile of the fat tissue, a custom-built SD-OCT system was used. As shown in Figure 3, the OCT system utilizes a superluminescent diode (SLD, EXALOS AG, Schlieren, Switzerland, central wavelength = 855 nm, bandwidth = 55 nm, output power = 5 mW) as a broad-band light source, a spectrometer unit comprising a diffraction grating (1200 L/mm @ 840 nm; Wasatch Photonics, Inc. Durham, NC, USA), a focusing lens, and a line CCD camera (E2V, AVIIVA, Chelmsford, UK). The sample arm of the fiber coupler was connected to a sample probe unit consisting of a fiber collimator lens (Thorlabs, Inc.), two single-axis galvanometric scanners, and single achromatic doublet as a scanning lens. The optical power on the tissue sample is measured to be 1.3 mW. The reference arm of the fiber coupler is attached to an OCT reference arm unit comprising a fiber collimator lens (Thorlabs, Inc.), an achromatic doublet lens, a neutral density filter (Newport, Irvine, CA, USA), and a AR-coated mirror. The OCT system has theoretical axial resolution of ~6 μm, lateral resolution of ~10 μm (FWHM), and sensitivity of ~100 dB.

Our imaging system acquired single A-lines in 25 microseconds and complete cross-sectional images comprising 520 A-lines at a rate of 40 frames per second. In this system, the acquired OCT images from the tissue sample were 12 mm × 8 mm and the imaging range was 3.5 mm. The OCT B-scans of the ablated tissue with different radiant exposure times (7, 15, 30, and 60 s) were acquired and processed at the lateral position which has the maximum ablation depth [26].

### 2.4. In Vitro Tissue Mass Loss Measurements

**Figure 4:**
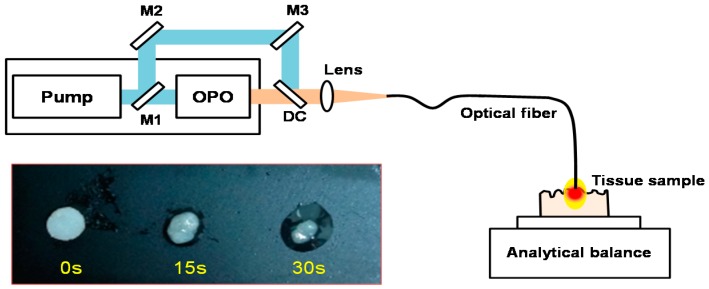
Experimental setup for tissue ablation measurement. Adipose tissue samples were placed on the pan of analytical balance. The laser was guided using a flat-cleaved 400 μm multimode fiber. As the tissue changed its phase, liquified adipose tissue was immediately removed and the mass change was recorded. Inset shows typical tissue phase changes with different ablation time at 1980 nm, 1 W. OPO: OPO cavity, M#: mirror, DC: dichroic filter.

An *in vitro* experiment was conducted to quantify the wavelength dependent adipose tissue removal rate. Figure 4 shows the experimental setup for *in vitro* tissue ablation. Ablation wavelengths from pump (1064 nm) and OPO cavity (1980 and 2300 nm) were selected using a movable mirror (M1) and dichroic filters (DC). The focused laser was delivered to the tissue sample using a flat-cleaved 400 μm multimode fiber.

Punch biopsy of adipose tissue with an initial mass of ~2.5 g was harvested from fresh porcine fat tissue. Tissue was mounted and ablated on the pan of a micro-balance. Liquefied fat was removed using absorption fabric after laser ablation. The tissue mass was recorded every 20 s after removing phase changed tissue. To minimize the mass loss from water evaporation by the spontaneous air circumstance, the experiment was conducted in a high humidity condition. Least squares regression was used for data analysis and calculation of the tissue ablation rate after laser irradiation with pump, signal, and idler wavelengths.

## 3. Results

**Figure 5:**
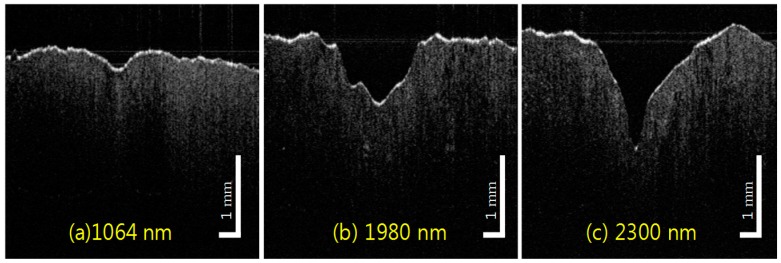
Representative OCT images of craters from laser irradiation at wavelength 1064 nm (**a**); 1980 nm (**b**); and 2300 nm (**c**) with 15 s ablation time. These particular images resulted from laser irradiation using a 400 micron multimode silica fiber at 10 W for pump (total energy delivery of 119.4 kJ/cm^2^) and 1 W for signal and idler (total energy delivery of 11.9 kJ/cm^2^).

Figure 5 shows representative OCT B-scans from the ablated fat tissue at a radiant exposure of 5 W (for pump) and 1 W (for idler and signal irradiation). The 2300 nm wavelength displayed the largest ablation depth (capability) and created a narrow elongated crater for a deeper tissue area, whereas 1980 nm irradiation wavelength created a relatively wide crater with a reduced ablation depth. These two frequency-converted wavelengths show superior ablation capability when compared with the non-converted pump wavelength of 1064 nm. In the latter case, 1 W exposure is far below the ablation threshold; no evident tissue ablation is observed at 1 W irradiation. For quantitative analysis of the OCT images, OCT B-scan is recorded at the deepest region of each ablation crater at same radiant exposure for three different wavelengths of pump, signal, and idler. The maximum ablation regions are decided by laterally scanning whole regions of ablation crater. The crater depth and width are measured from 7 to 60 s with 5 W (1064 nm) and 1 W (1980 nm, 2300 nm) average output power.

**Figure 6:**
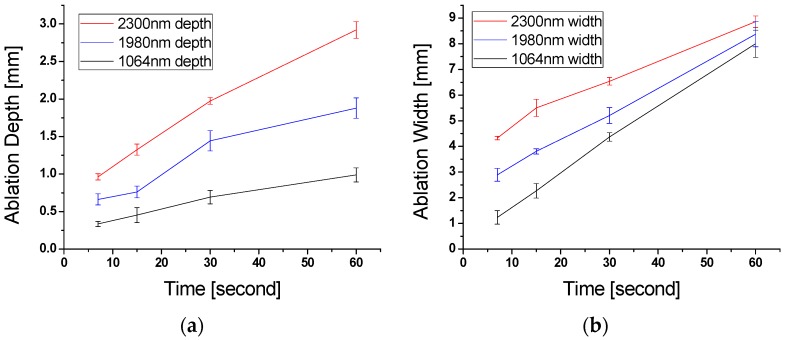
Ablation dimension measurement using OCT after laser irradiation at pump, signal, and idler wavelengths. Crater depths (**a**) and diameters (**b**) in millimeters versus laser irradiation time.

Figure 6 shows laser irradiation energy dependent crater depths and diameters at pump, signal, and idler wavelengths. Crater depths at 2300 and 1980 nm rapidly increase in nearly non-linear fashion as the incident time increased. However, the crater depth at 1064 nm increases slowly over the same energy irradiation. The results from crater size measurement after laser irradiation at three different wavelengths (pump, signal, and idler) with adipose tissue samples reveal significantly enhanced lipolysis effects achieved by the nonlinear frequency conversion laser system with chromophore targeting at the near 2 μm region.

**Figure 7:**
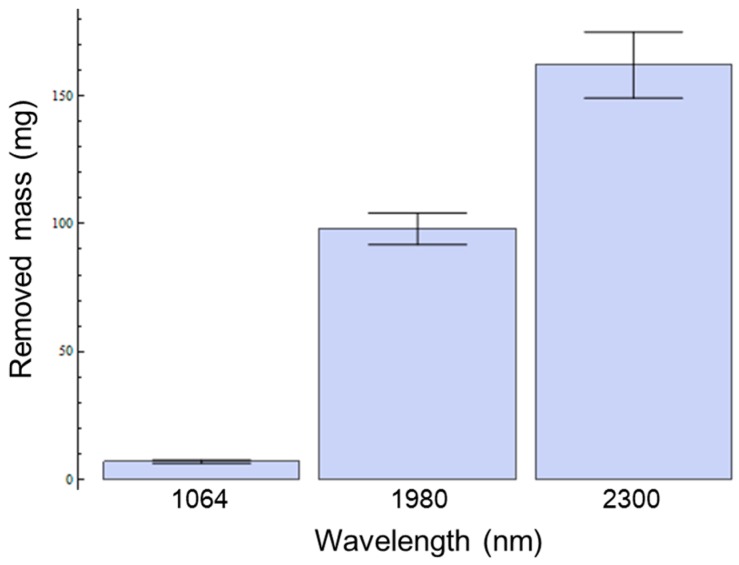
Amount of phase changed masses after laser irradiation at 79.5 kJ/cm^2^ for pump, signal, and idler (1064, 1980, and 2300 nm).

Figure 7 depicts measured reduced mass of tissue samples with three different laser irradiation wavelengths. For the quantitative evaluation of *in vitro* tissue mass losses after laser irradiation, the measured mass losses for signal and idler at a radiant exposure of 79.5 kJ/cm^2^ show that the highest mass removal for adipose tissue is achieved at idler (2300 nm) wavelength. From least square regression, the calculated tissue ablation rates are 19.92 mg/min (1064 nm, 5 W), 48.84 mg/min (1980 nm, 1 W), and 79.5 mg/min (2300 nm, 1 W).

## 4. Discussion

**Figure 8:**
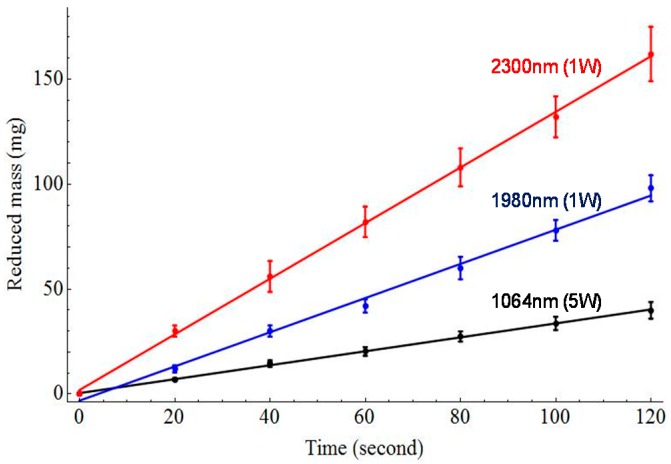
Measured reduced mass of tissue samples with different laser irradiation times for pump, signal, and idler wavelengths. The points represent the experimental values and the solid lines are linear regression fits (n > 10).

Comparison studies including pump, and frequency converted signal, and idler wavelengths demonstrated significantly enhanced lipolysis effects with increasing wavelength specificity for the adipose tissue in Figure 7. Phase changed mass loss after laser irradiation at 79.5 kJ/cm^2^ for three different wavelengths was measured to be 6.64 ± 0.62 mg, 78.00 ± 4.93 mg, and 131.99 ± 9.78 mg, for 1064, 1980 and 2300 nm, respectively. In Figure 7, frequency converted wavelengths of signal and idler require 22 and 38 times less energy, respectively, than the 1064 nm to lipolysis the same amount of adipose tissue. The ablation rate of 2300 nm wavelength had 1.63 times greater ablation efficiency (76 mg/min) than 1980 nm with same average power (1 W) shown in Figure 8.

However, the 2300 nm laser induced tissue carbonization especially when the tissue was exposed to long laser irradiation time. This is the reason that the laser system was operated as a quasi-CW laser and attributed to high absorption for both water and fat. The role of temporal characteristics (*i.e.*, pulse duration) of laser system is important to achieve efficient tissue ablation and minimal thermal damage. To minimize collateral damage, higher peak power is preferred because the thermal energy is consumed for tissue phase transformation before it diffuses into surrounding tissues by a single intense pulse. Although typical laser lipolysis is performed by inserting a cannula into the subcutaneous fat layer under the skin as well as there is a low probability of thermal damage to the unintended tissue layer, thermal effect control is an important issue for safer and minimally invasive laser tissue ablation. Due to high optical absorption differences between water and fat, we expect the 1980 nm have the potential selective photothermolysis effect, especially for tissues with high water content and low fat ratio.

We observed almost same ablation efficiency between 1 W/1980 nm and 10 W/1064 nm (48.8 and 44 mg/min). It is notable that we used relatively small core (400 μm) bare-cleaved multimode fibers for the experiments which have a small interaction area with tissue samples with minimized power (1 W for signal and idler) [27]. However, the achieved ablation efficiency was remarkable and effective adipose tissue phase change could be achieved with relatively small power and irradiation time. Here, frequency converted wavelengths potentially facilitate time-effective surgical treatment and cost-effective laser system scale downing with enhanced user safety. Moreover, the OPO-based nonlinear frequency conversion process provided precise wavelength tuning by controlling quasi phase matching condition in the nonlinear crystal (*i.e.*, crystal temperature, crystal position) which enables precise chromophore selecting and targeting in biological tissues. Future introduction of diffusing fiber geometry (side emitting) will enable further increment of the tissue removal efficiency by dramatically increasing laser-tissue interaction areas with the widespread and cost-effective 1064 nm pump laser and the proposed OPO system. We expect this system has a potential to be commonly used in user-friendly desktop environment for laser lipolysis treatment.

## 5. Conclusions

An optical parametric oscillator (OPO)-based 2 µm laser system was developed and its adipose tissue ablation efficiency was investigated. The wavelength dependence measurements of laser lipolysis effect were evaluated using different lasers with before and after the nonlinear frequency conversion: 1064 nm *vs.* 2300 and 1980 nm wavelengths. The experimental results demonstrated that the greatest ablation crater size and mass removal in adipose tissue was achieved at the 2300 nm wavelength followed, in order, by 1980 and 1064 nm. Quantitatively, frequency converted wavelengths of signal and idler consume 22 and 38 times less energy than the non-converted pump wavelength, 1064 nm when removing the same amount of adipose tissues. Adipose ablation was highly wavelength dependent. Additionally, the mid-infrared laser source 2300 nm enabled the largest craters and greatest amount of mass removed at all radiant exposures. Therefore, 2300 nm and 1980 nm wavelengths provided the higher ablation efficiency as compared with the pump 1064 nm laser.
